# Twin births, sex of children and maternal risk of ovarian cancer: a cohort study in Norway

**DOI:** 10.1038/sj.bjc.6603687

**Published:** 2007-03-27

**Authors:** G Albrektsen, I Heuch, S Thoresen, G Kvåle

**Affiliations:** 1 Department of Mathematics, University of Bergen, Johannes Brunsgate 12, 5008 Bergen, Norway; 2 Cancer Registry of Norway, Montebello, 0310 Oslo, Norway; 3 Section of Pathology, Gade Institute, University of Bergen, Haukeland University Hospital, 5020 Bergen, Norway; 4 Centre for International Health, University of Bergen, Armauer Hansens hus, 5020 Bergen, Norway

**Keywords:** ovarian cancer, histology, twin births, sex of children

## Abstract

In a follow-up of 1 208 001 women aged 20–74 years, no significant association was found between twin births (112 cases) and risk, though those with twin girls had a non-significantly higher risk than those with singleton births; among the latter, those with girls only had a higher risk of endometrioid tumours (incidence rate ratio 1.35; 95% confidence interval 1.03–1.76, based on 475 cases) than women with boys only.

The protective effect of pregnancies on the risk of epithelial ovarian cancer is usually explained by their influence on lifetime number of ovulations (Risch, 1998). Women prone to have multiple births have a higher level of follicle-stimulating hormone (Bulmer, 1970; Corney *et al*, 1981; Risch, 1998) and may also ovulate more often (Allen, 1981), possibly leading to an increased risk (Risch, 1998; Lukanova and Kaaks, 2005). Certain maternal or pregnancy-related factors associated with twinning may however be related to a reduced risk (Risch, 1998) though only a slightly lower or similar risk has been reported in twin compared to singleton mothers (Wyshak *et al*, 1983; Olsen and Storm, 1998; Lambe *et al*, 1999; Whiteman *et al*, 2000; Titus-Ernstoff *et al*, 2001; Neale *et al*, 2004, 2005).

Hormones may play a role in ovarian cancer carcinogenesis (Riman *et al*, 1998; Risch, 1998; Lukanova and Kaaks, 2005). Maternal hormone levels have been found to be higher in pregnancies involving twins (TambyRaya and Ratnam, 1981; Risch, 1998; Storgaard *et al*, 2006). The levels of oestrogens, androgens and human chorionic gonadotropin (hCG) seem to differ by sex of the fetus in singleton gestations (Robinson *et al*, 1977; Forest *et al*, 1980; Haning *et al*, 1989; Cerhan *et al*, 2000; Luke *et al*, 2005). A different risk pattern according to sex of children may therefore throw light on alternative hormone-related hypotheses, but few studies have focused on this issue.

We have examined associations between twin births, sex of children and the risk of ovarian cancer in a large cohort of parous Norwegian women.

## MATERIALS AND METHODS

The present study includes Norwegian women born in the period 1925-1979, as recorded in the national population register. The study population comprised 1 208 001 parous women, contributing a total of 27 634 403 person-years at risk in the age interval 20–74 years during follow-up until 1st January, 2000. The mean follow-up time was 22.9 years, range 1 month–45 years. A total of 28 911 women had experienced a twin birth; 354 of these had two twin births and five had three. Women with higher plurality of a multiple birth (*n*=461) were excluded. Information on reproductive history (date of birth for each live born child) and cancer diagnoses was assessed through national registers.

A total of 5606 women were diagnosed with ovarian cancer (ICD 7th Revision, code 175). Of these, 5204 (92.8%) were classified as invasive epithelial ovarian cancer, 291 (5.2%) as non-epithelial ovarian cancer, whereas 111 (2.0%) had no valid histological code. Borderline tumours were not considered. Separate analyses were performed for epithelial and non-epithelial ovarian cancer, and by histological subtype (Table 1). A woman was withdrawn from the risk set at date of diagnosis of any type of ovarian cancer.

### Statistical analysis

A log-linear Poisson regression model of person–years at risk (Breslow and Day, 1987) was applied. Attained age was considered as timescale (categorical variable, 1-year intervals). The variables representing parity (number of full term births) and different birth characteristics were treated as time-dependent categorical variables. A twin birth was identified through identical birth dates of siblings. Birth-cohort, maternal age at first and last (most recent) childbirth, were all considered as categorical variables in 5-year intervals.

Tabulation of individual records into person–years tables and Poisson regression analyses of person–years at risk, with calculation of maximum likelihood estimates of incidence rate ratios (IRR) and likelihood ratio tests were performed by means of the EPICURE programme package (Preston *et al*, 1996).

## RESULTS

No significant association was found between twin births and risk of epithelial ovarian cancer (112 cases), but women with twin girls had a slightly higher risk than women with singletons only, whereas women with twin boys or twins of both sexes, had a slightly lower risk (Table 2). The elevated risk associated with twin girls was more pronounced before age 45 years, but the interaction test did not reach statistical significance (Table 2).

The association with sex of twins reached statistical significance for mucinous tumours (*P*=0.019, test for heterogeneity); women with twin girls had almost twice the risk of singleton mothers (IRR=1.67, 95% confidence interval (CI)=0.98–2.91, 13 of 20 cases), whereas with twin boys the risk was lower (IRR=0.26, 95% CI=0.07–1.04; two cases only). An adverse effect of twin girls was also seen for serous tumours before age 45 years (IRR=2.08, 95% CI=1.15–3.77 and IRR=0.72, 95% CI=0.40–1.29 in women below and above age 45 years, respectively; *P*=0.10, test for interaction). Twin mothers had a slightly reduced risk of endometrioid tumours (IRR=0.52, 95% CI=0.23–1.16); numbers were too low to discriminate by sex of the twins (Table 1). Twin mothers had a higher risk of non-epithelial ovarian cancer (IRR=1.79, 95% CI=0.98–3.28), especially stromal tumours (IRR=3.97, 95% CI=2.00–7.86).

In women with singleton births, no association was found between sex of children and the risk of epithelial ovarian cancer (Table 2). However, women with girls only had significantly higher risk of endometrioid tumours than women with boys only (IRR=1.35, 95% CI=1.03–1.76), whereas women with both girls and boys had a rather similar risk (IRR=1.16, 95% CI=0.88–1.52). The adverse effect of having girls only was more pronounced in women with at least three children (IRR values of 1.34, 1.28 and 1.61 in women with one, two and ⩾three children, respectively), and in women above age 45 years (IRR values of 1.23 and 1.40 in women below and above age 45 years, respectively). No consistent association was seen between sex of children and other histological subtypes (results not shown).

## DISCUSSION

This large cohort study did not show any overall association between twin births and the risk of epithelial ovarian cancer, but women with twin girls seemed to have a higher risk than singleton mothers. The increased risk was particularly pronounced for mucinous tumours, and serous tumours before age 45 years; a slightly lower risk was seen at older ages. Sex of children in singleton births was associated with the risk of endometrioid tumours only; women with girls only had the highest risk.

Previous studies have found a lower or rather similar risk of epithelial ovarian cancer in twin compared to singleton mothers (Olsen and Storm, 1998; Lambe *et al*, 1999; Whiteman *et al*, 2000; Titus-Ernstoff *et al*, 2001; Neale *et al*, 2004, 2005), but few have focused on sex of the twins, or performed subgroup analyses. One study based on pooled data from eight case–control studies (Whiteman *et al*, 2000) found an adverse effect of twinning in relation to mucinous tumours, but a protective effect in relation to non-mucinous tumours. However, no information was given on the sex of the twins. Another study (Lambe *et al*, 1999) found a more pronounced reduction in risk in mothers with twins of opposite sex than same-sex twins; no histology-specific results were presented.

Consistent with our finding for endometrioid tumours, one previous study (Gierach *et al*, 2006) found that women with girls only had higher risk of epithelial ovarian cancer than women with boys only, or boys and girls. In another study (Olsen and Storm, 1998), the sex ratio among newborns to mothers who later developed ovarian cancer was similar to that in infants with mothers without such a diagnosis.

There is growing evidence that oestrogens play a role in ovarian cancer carcinogenesis, as well as tumour progression (Mink *et al*, 2002; Cunat *et al*, 2004; Riman *et al*, 2004). The hCG level is higher during female than male gestations (Robinson *et al*, 1977; Haning *et al*, 1989; Cerhan *et al*, 2000; Luke *et al*, 2005) and hCG is involved in oestrogen production during pregnancy (Risch, 1998). Moreover, the hCG level has been found to be higher in twin than in singleton pregnancies (Haning *et al*, 1989; Luke *et al*, 2005), and highest when both twins are girls (Steier *et al*, 1989). A potential increased risk of mucinous and serous tumours in women with twin girls, and an increased risk of endometrioid tumours in women with girls only, may thus be related to an adverse effect of oestrogens, acting either at early or later stages of carcinogenesis during pregnancy. Oestrogen receptors have been found both in endometrioid, serous and mucinous ovarian tumours (Risch, 1998; Gadducci *et al*, 2004; Auranen *et al*, 2005; Lee *et al*, 2005), and also in normal ovarian surface epithelial cells (Cunat *et al*, 2004; Auranen *et al*, 2005). The apparent reduced risk of endometrioid tumours in twin mothers, however, is more likely explained by other, possibly maternal factors (Risch, 1998).

In summary, the present results suggest a complex relationship between twin births, sex of children and ovarian cancer risk, including a heterogeneity according to woman's age, as well as histological subtype. Women with twin girls or women with daughters only, seem to have an increased risk of certain histological types, whereas pregnancies involving a male foetus showed either no association, or a reduced risk. Maternal, as well as pregnancy-related factors, may explain these epidemiological findings. Further studies are needed to exclude the role of chance.

## Figures and Tables

**Table 1:** Distribution of histological subtype in ovarian cancer patients^a^, total and in twin and singleton mothers, respectively

	**Total**	**Twin mothers**	**Singleton mothers**
	**No. (%)**	**No. (%)**	**No. (%)**
*Epithelial*	5204 (92.8)	112 (88.2)	5092 (92.9)
Serous	2639 (47.1)	61 (48.0)	2578 (47.1)
Mucinous	1020 (18.2)	20 (15.7)	1000 (18.3)
Endometrioid	481 (8.6)	6 (4.7)	475 (8.7)
Clear cell	243 (4.3)	5 (3.9)	238 (4.3)
Other/unspecified adenocarcinoma	821 (14.6)	20 (15.7)	801 (14.6)
			
*Non-epithelial*	291 (5.2)	11 (8.7)	280 (5.1)
Stromal	108 (1.9)	9 (7.1)	99 (1.8)
Germ cell	109 (1.9)	1 (0.8)	108 (2.0)
Sarcomas	74 (1.3)	1 (0.8)	73 (1.3)
			
Unspecified	111 (2.0)	4 (3.1)	107 (2.0)
Total	5606 (100)	127 (100)	5479 (100)

a Aged 20–74 years at date of diagnosis.

**Table 2:** Incidence rate ratios (IRR with 95% confidence interval) of epithelial ovarian cancer by twin births and sex of the twins, and by sex of children in women with singleton births only^a^

	**No. of cases**	**P–year (× 10^4^)**	**IRR (95% CI)**	***P*-value^b^**
Twin birth				0.39
Never (singletons only)	5092	2706.2	1.00 (ref.)	
Ever	112	57.2	0.92 (0.76–1.11)	
*By sex of the twins* c				0.10
Two boys	33	19.3	0.82 (0.58–1.16)	
Two girls	49	19.4	1.21 (0.91–1.61)	
Boy and girl	30	18.5	0.73 (0.51–1.05)	
*Within age groups (year)*				
20–44				
Two boys	9	12.2	0.83 (0.43–1.60)	
Two girls	20	12.3	1.83 (1.18–2.85)	
Boy and girl	9	11.2	0.88 (0.46–1.69)	
45–54				
Two boys	11	41.5	0.76 (0.42–1.38)	
Two girls	12	41.5	0.83 (0.47–1.46)	
Boy and girl	8	41.7	0.55 (0.27–1.10)	
55–74				
Two boys	13	29.5	0.88 (0.51–1.51)	
Two girls	17	29.4	1.13 (0.70–1.83)	
Boy and girl	13	31.6	0.81 (0.47–1.39)	
*P-value, test for interaction*^d^			0.20	
				
Singleton births, sex of children				0.96
Boys only	1163	662.9	1.00 (ref.)	
Girls only	1339	735.7	1.02 (0.94–1.11)	
Boys and girls	2590	1298.3	1.00 (0.92–1.08)	

a Results based on Poisson regression analysis of person–years (P–year) at risk, adjusted for attained age, birth-cohort, number of births, and maternal age at first and most recent birth.

b Test for heterogeneity.

c Women with singleton births as reference group.

d Test for heterogeneity in risk estimates in women above and below age 45 years.
